# Engagement of Government Social Media on Facebook during the COVID-19 Pandemic in Macao

**DOI:** 10.3390/ijerph18073508

**Published:** 2021-03-28

**Authors:** Patrick Cheong-Iao Pang, Qixin Cai, Wenjing Jiang, Kin Sun Chan

**Affiliations:** 1 Victoria University Business School, Victoria University, Melbourne 3000, Australia; mail@patrickpang.net; 2 Institute of Social Security, School of Public Administration and Policy, Renmin University of China, Beijing 100872, China; alan.cai.qixin@ruc.edu.cn; 3 Department of Government and Public Administration, Faculty of Social Sciences, University of Macau, Macao; kschan@umac.mo

**Keywords:** government, social media, Facebook, content analysis, Macao, communication, engagement, COVID-19

## Abstract

Government social media is widely used for providing updates to and engaging with the public in the COVID-19 pandemic. While Facebook is one of the popular social media used by governments, there is only a scant of research on this platform. This paper aims to understand how government social media should be used and how its engagement changes in prodromal, acute and chronic stages of the pandemic. We collected 1664 posts and 10,805 comments from the Facebook pages of the Macao government from 1 January to 31 October 2020. Using word frequency and content analysis, the results suggest that the engagement was relatively low at the beginning and then surged in the acute stage, with a decreasing trend in the chronic stage. Information about public health measures maintained their engagement in all stages, whereas the engagement of other information was dropping over time. Government social media can be used for increasing vigilance and awareness in the prodromal stage; disseminating information and increasing transparency in the acute stage; and focusing on mental health support and recovery policies in the chronic stage. Additionally, it can be a tool for controlling rumors, providing regular updates and fostering community cohesion in public health crises.

## 1. Introduction

In this ongoing coronavirus disease 2019 (COVID-19) global pandemic, government social media plays an important role in engaging the public to combat the disease. A tremendous public health risk such as the COVID-19 pandemic shares a lot of common characteristics with other crises, for example, a high level of uncertainty, unexpected rapid development and short triggering events [1]. Therefore, communication efforts need to be ramped up to match the level of risks. Apart from the adverse effects of an “infodemic” (i.e., misinformation and panic being widely spread on social media) [2,3], research highlights the potential of social media to provide trusted information and counteract misinformation [4,5], cultivate information security behavior against scams [6], raise awareness and promote behavioral changes [7]. Despite the growing body of research in this space, only a limited number of studies (for example, [8,9,10]) have investigated the use of social media by governments and public health authorities. As such, our study aims to conceptualize the use of government social media in this COVID-19 crisis for its effective use in future public health crises.

Our analysis of the literature shows its imbalance in both the choices of social media platforms and the period of data collection. Many studies used Twitter (e.g., [11,12,13]) and Weibo (e.g., [9,14,15,16,17]) as data sources, and only a scant amount of research examined the content on Facebook (e.g., [8,18]). Due to the different use, culture and politics of different social media platforms [19], Facebook users may pose different behaviors and this motivates our work. In addition, our literature review finds that most of the social media research about COVID-19 used data between December 2019 and April 2020. While this is understandable as user reactions were most intense at the early stages, however, as many countries move forward to another phase of the pandemic, people are expected to react differently both online and offline in the new normal. Therefore, our study can address these blanks and shed light on the use of government social media at the beginning of the end of this global pandemic.

The dynamics of the COVID-19 pandemic often requires quick responses and swift decision making, which are similar to the handling of public health crises. As such, we use the perspective of crisis management that offers a systematic framework to understand how government social media is used at different points of time, which is important since social media users prefer different content and disseminate information differently in different stages of public health crises [20,21]. More specifically, the crisis model proposed by Fink divides a crisis into four stages [22], which allows us to compare the use of government social media in response to COVID-19 separately.

Despite having the world’s highest population density [23], the Macao Special Administration Region (abbreviated to Macao SAR or Macao) is one of the jurisdictions in the world which has successfully eliminated the transmission of the disease. As a popular destination of tourists, Macao welcomes 38 million tourists per year, and thus any ineffective infectious disease control can be catastrophic [24]. With rapid early responses and stringent preventive measures [24], the city has recorded more than 350 days without local community transmission at the time of writing (March 2021). As the community has entered the recovery stage, the case study of Macao not only provides understandings about their use of government social media, but also an insight at how the engagement of social media can be leveraged in the subsequent stages of the pandemic. This knowledge will be useful for Macao and other places to handle public health crises in the future.

The content analysis methodology was mainly used in this study. We collected 1664 COVID-19-related posts and 10,805 comments of these posts from the official Facebook pages of the Macao SAR government from 1 January 2020 to 31 October 2020 with software. These posts were classified based on their nature with a qualitative approach, whereas an exploratory word frequency analysis was performed on the comments. In addition, we examined the trends of different types of engagement associated with the posts, for example, total, positive and negative engagement, as well as the numbers of comments and shares. We also conceptualize the findings and propose the potential applications of government social media for future public health crises.

The rest of this paper is structured as follows. Firstly, we give a brief introduction of the COVID-19 outbreak in Macao and the literature review that supports our work. Then, we present our data collection details, analysis methods and results, followed by the discussion of the main findings and their implications. Finally, we conclude the article with the key messages identified in our work.

## 2. Background: The COVID-19 Outbreak in Macao

The emergency and crisis management system in Macao originated in the severe acute respiratory syndrome (SARS) outbreak in 2003 [25]. This system and key legislations such as the Law on Prevention, Control and Treatment of Infectious Diseases and the 10-Year Plan for Disaster Prevention and Mitigation of the Macao SAR (2019–2028) have been established to empower the government to rapidly respond to mass emergencies and disasters. Before COVID-19, the crisis management system was primarily used for natural disasters such as typhoons Hato (2017) and Mangkhut (2018) [25]. In this current outbreak, the system and the legislation enables the government to realize public health measures and isolate potentially infectious patients [24].

In the early days, Macao put some preparatory measures in place even before the start of the local outbreak [24], in response to the cases of pneumonia of unknown cause in Wuhan, China at the end of December 2019 [26]. Starting from 1 January 2020, the checking of body temperature was enforced for all passengers travelled from Wuhan, and health declaration forms became mandatory in order to enter Macao. The government established a 24-h COVID-19 Coordination Center for monitoring the development of the potential infections on 21 January [24].

On 22 January, a traveler from Wuhan was identified as the first confirmed infection in Macao [27]. In the following two weeks, nine more cases were found and admitted to hospital [28]. In the meantime, various stringent measures (Table 1), including stricter border control, centralized procurement and the mandatory use of face masks, were introduced to reduce the transmission of the virus. Casinos and border limitations were enforced to control the outbreak with a huge cost to the tourism and gaming industries [23,29]. Local transmissions continued to be discovered until 29 March [30].

After the elimination of local community transmission, Macao recorded only three imported cases [27], and has entered the phase of recovery. While strict preventive measures largely remained, the government has been focusing on reviving the economy, protecting job opportunities and supporting businesses (for instance, two rounds of economic assistance measures, the subsidization of water and power bills, tax reduction and exemption, etc.). The city also saw the ease of some border control measures and schools resumed classes in stages.

In summary, the Macao SAR government has suppressed the COVID-19 outbreak with a combination of various policies. Many of these policies were also posted to different social media platforms, including Facebook.

## 3. Literature Review

### 3.1. Crisis Management and COVID-19

Crises are unpredictable and highly uncertain events in an organization or even a society [31]. Its unpredictable and disruptive nature lead to the needs for crisis management, which aims to identify the key issues in a crisis and reduce harm [32]. Among the research of crisis management, the four-stage model proposed by Fink is a popular tool for researchers to understand the development of crises [22]. This model divides the lifecycle of a crisis into four crisis stages [33,34], namely prodromal, acute, chronic and resolution. In this work, we combined these stages with the progress of the COVID-19 pandemic based on the time when cases were confirmed. Table 2 lists our definitions of these stages and explains how this model can split the timeline of the COVID-19 outbreak for further analysis.

### 3.2. Government Social Media and COVID-19

Social media applications can be strategically applied to foster an open government and to improve citizen–government interaction [37]. Previous studies have highlighted the importance of the engagement of government social media to facilitate the communication between the public and governments [38,39,40,41]. Some scholars have also identified challenges, risks, organizational barriers and cultural differences with government social media [39,42]. In certain settings, Facebook is preferred to other social media platforms as a means of participating in local government issues [40], which may imply that Facebook is a critical tool for effective communication between the public and governments during public health emergencies. Government Facebook pages can attract users to share and disseminate public health messages [8]. A recent study suggests that a communication strategy is needed for public health authorities to enhance the preparedness and the responses in public health crises [10].

There has been a growing body of research regarding the use of social media during COVID-19. As the electronic word of mouth, online information such as reviews and social media posts can change people’s behavior [43,44,45], and therefore misinformation and fake reviews are issues in controlling the pandemic. Some scholars have expressed concerns about the difficulty of building trust amid misinformation spread on social media [46]. Others acknowledge the potential of social media for positive purposes [47]. Additionally, studies show that many categories of information were posted on social media. To synthesize the relevant literature, we list these findings in Table 3, which serve as the theoretical foundation for the classification of our data collected from Facebook.

In this article, we aim to contribute to the understanding of how the engagement of government social media, particularly Facebook, evolves in the development of the pandemic. As such, the following research questions were formulated for this study:

- RQ1: How does public’s engagement of government social media change over the course of the COVID-19 pandemic?
- RQ2: How do the categories of the content posted by a government affect the level of such engagement?
- RQ3: What are the implications for governments to use social media to engage with the public in pandemics?

## 4. Methods and Data Collection

### 4.1. Data Collection

The information posted on the official Facebook pages of the Macao SAR government was the subject of this study. According to the annual internet usage report published by the Macao Association of Internet Research, Facebook is the second most popular social networking platform used by Macao residents [52]. In 2020, 69% of Macanese internet users used Facebook, among them 94% were 18–34 years old, 75% were 35–54 years old, and 36% were over 55 [52]. Facebook is one of the social networking platforms used by the government to engage with the public since 2017 [53], and therefore this platform can provide a reasonable sample for us to study the use of government social media in this pandemic.

Customized scripts written in Python (version 3.8) were used to crawl data from Facebook every three days. Post content, comments and emotions were updated in our database on a rolling basis to allow for the capture of late interactions from other Facebook users even if the posts had been published for some time. Human ethics applications were exempted by the University’s committee because we used publicly accessible data and would report the results in an aggregated manner.

### 4.2. Methods

Our research design followed the qualitative content analysis approach which allows for the study of complex phenomena and exploration of patterns in the health context by transforming a large amount of textual data into systematic themes and categories [54]. Content analysis is also used in social media analysis during the pandemic [55]. We used qualitative axial coding [56,57] to sort Facebook posts into a number of categories based on their text content. Both deductive and inductive reasoning was used in the coding process. A preliminary codebook was deducted from the existing literature of the content of social media during crises (Table 2) and this codebook was used to guide the analysis. In this process, codes that did not exist in the codebook were inducted through re-reading the data and merging similar codes. This approach can preserve the theories and principles that are applicable for our analysis, while allowing for the flexibility for unexpected or new insights to be captured, which results in a rigorous, comprehensive and reliable study [58].

**Figure 1:**
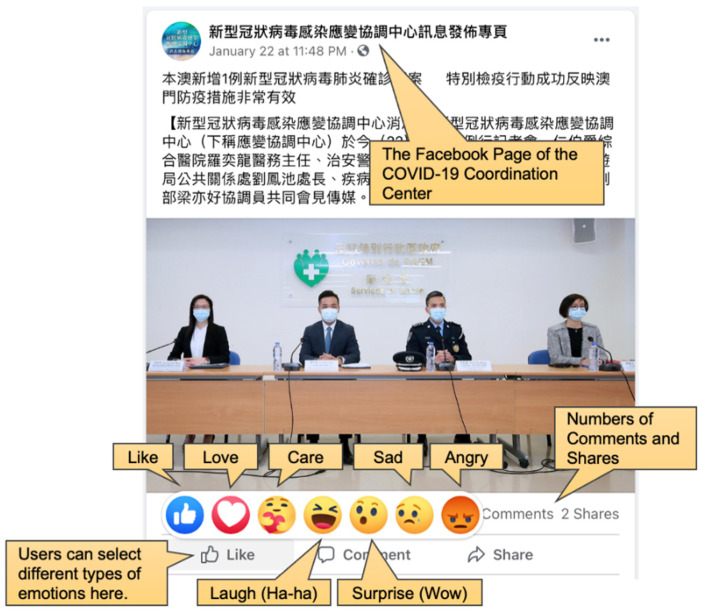
A sample COVID-19 Facebook post authored by the government.

We also obtained the numbers of comments, shares, likes and other emotions. As illustrated in Figure 1, in addition to “likes”, modern Facebook allows users to select other emotions, such as love, angry and sad. This information can be used for measuring the level of different types of engagement of government social media. For the purposes of this study, we categorized these choices of engagement into the following four metrics:

- Positive emotions: The sum of likes, love, laugh and care emotions
- Negative emotions: The sum of angry and sad emotions
- Numbers of comments
- Numbers of shares

Positive and negative emotions were used for understanding the sentiment expressed by Facebook users; the numbers of comments showed the degree to which users wanted to interact with the government and other users by leaving comments; and the numbers of shares indicated the magnitude of the propagation of information. The “surprise” emotion was excluded because it could not represent neither positive nor negative. The average values of such variables were then aggregated by calendar weeks in order to observe the trends at different times during the pandemic. In addition, we compared these figures across different post categories and crisis stages.

The content of user comments can reflect their areas of concerns, but such content can be noisy for analysis, for example, someone may comment to the original post, reply to other users, or even disseminate non-relevant messages. In this work, we performed a word frequency analysis to explore the topics that most people commented under the posts. We utilized a word segmentation library PKUSEG (version 0.25) created by Peking University [59] to split Chinese sentences into words, then the NLTK toolkit (version 3.4.5) [60] was applied to calculate the occurrences of each word within the data. The most used 10 words (threshold determined by our previous work [61]) were examined to identify users’ interests and concerns.

## 5. Results

### 5.1. Data Overview

Before the COVID-19 outbreak in Macao, there were no dedicated government Facebook pages about public health. We targeted the official government Facebook pages (e.g., Macao SAR Government News) and manually selected posts about the disease. After the establishment of the COVID-19 Coordination Center and its dedicated page, information related to COVID-19 was posted on this specific page and therefore only the posts on this page were included in our research. We totally collected 1664 posts from Facebook, including text-based posts, images and recorded live videos in a 304-day duration from 1 January 2020 to 31 October 2020. After removing comments without text content (e.g., containing only emojis and images), 10,805 comments of these posts were also retrieved. In average, the government authored 5.47 COVID-related posts per day and users posted 6.49 comments per post. These posts were assigned into three different crisis stages using the criteria outlined in Table 2, and the cut-off dates of stages are listed in Table 4. Then, two researchers were trained to perform coding on the data. Their coding achieved 87% of agreement, and the inter-coder reliability Krippendorff’s Alpha α = 0.810 which was over the threshold value (α ≥ 0.800) required for the use in academic research [62,63].

### 5.2. Overall Trends

**Figure 2:**
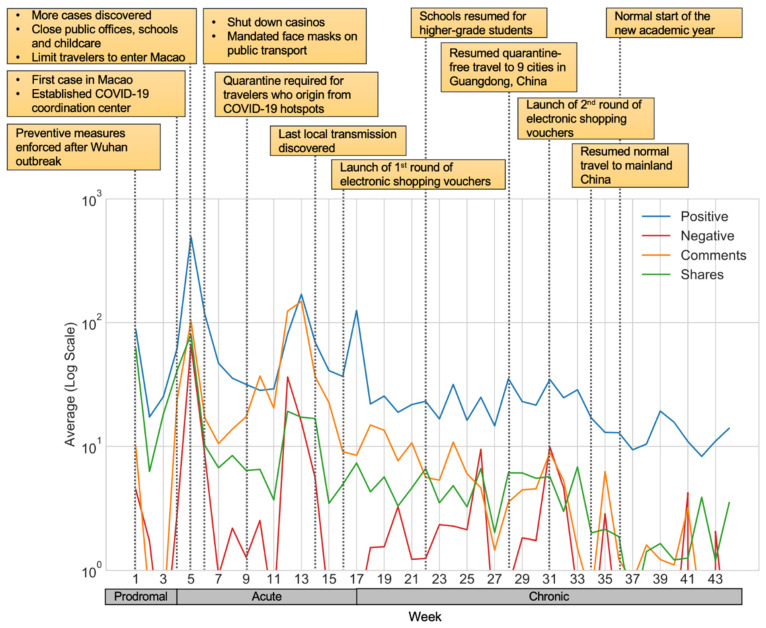
Trend of engagement and COVID-19 key events in Macao.

Firstly, we analyzed the engagement in the prodromal, acute and chronic stages, respectively. Figure 2 displays the trends of the weekly average values of the metrics of Facebook engagement in a logarithmic scale, shown along with the key events of the outbreak in Macao. In the first few weeks of the prodromal crisis stage, the engagement could be observed to go downwards initially and gradually turned upwards when the time got closer to the discovery of the first COVID-19 case. Since the first confirmed case, positive emotions, comments and shares increased to a higher level in the acute crisis stage, accompanied by occasional surges of negative emotions. As the pandemic entered the chronic crisis stage, all metrics tended to decrease over time. Overall, the level of positive emotions was higher than the negative ones in the entire pandemic. For the numbers of comments and shares, they both demonstrated greater values in the acute stages. Generally speaking, the overall engagement showed a decreasing trend after the earlier weeks in the acute stage.

Table 5 shows the descriptive statistics of the engagement of the public throughout the three stages in the outbreak. Although the chronic stage included the most posts, the engagement was most intense in the acute stage, demonstrated by the higher means of various metrics. In addition, some emotions have a high standard deviation or large maximum values (for instance, the positive emotion and the number of comments in the acute stage), which shows that there are outlier posts that attracted extreme levels of engagement than other posts.

**Figure 3:**
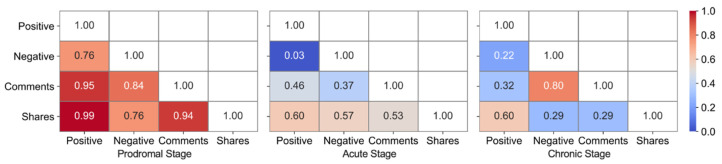
Correlation matrix of engagement in three stages.

Figure 3 presents the Pearson correlation matrix of engagement in the three crisis stages, and each stage demonstrates different patterns across the engagement metrics. In the prodromal stage, positive emotions were highly correlated to the numbers of comments and shares, and comments and shares were also strongly correlated to each other. In the acute stage, there were no obvious correlations among these types of engagement. However, in the chronic stage, negative emotions showed a moderate level of correlation to the number of comments.

### 5.3. Post Categories

We investigated how the engagement of categories of content differs in various stages of the pandemic. As abovementioned, our data underwent a qualitative coding process, and they were grouped into seven categories, as shown in Table 6. The first six categories matched to the concepts reported in other literature, while the last category (Press Conference Live) was derived from our data. An example post translated into English is given for each category in the table.

### 5.4. Trends of Post Categories

**Figure 4:**
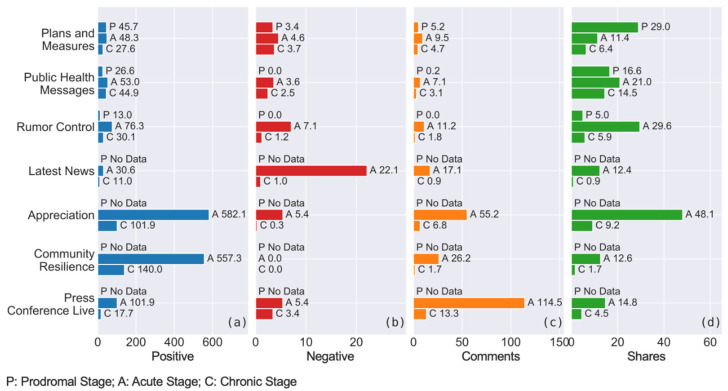
Comparison of the engagement of different post categories in three stages: (**a**) positive engagement; (**b**) negative engagement; (**c**) numbers of comments; (**d**) numbers of shares.

Figure 4 shows the trends of the engagement attracted by the above post categories, denoted by the average numbers of positive emotion, negative emotion, the numbers of comments and the numbers of shares in different stages of the pandemic.

The data illustrate that two post categories, namely Plans and Measures and Public Health Messages, could maintain similar levels of positive emotions, negative emotions and comments throughout all three stages, while other categories showed downward trends after the acute stage.

Additionally, for Appreciation and Community Resilience posts, it is notable that they could attract a high degree of positive engagement and more shares in the acute stage, but they recorded a large decline in the subsequent chronic stage. Similarly, Press Conference Live videos collected a substantial number of comments and shares, as well as relatively strong positive engagement during the acute stage, however, these figures dived in the later stage too.

For the number of shares (Figure 4d), we can observe that they were generally high in the middle stage and relatively low in the chronic stage. This pattern was particularly obvious for Rumor Control and Appreciation categories.

### 5.5. Content Analysis of Three Crisis Stages

**Figure 5:**
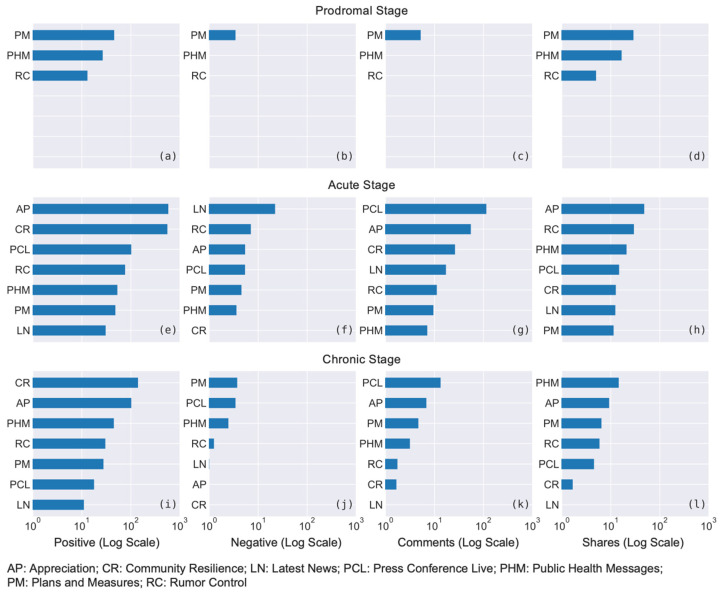
Averages of engagement in three stages ordered by post categories: (**a**) positive engagement in the prodromal stage; (**b**) negative engagement in the prodromal stage; (**c**) numbers of comments in the prodromal stage; (**d**) numbers of shares in the prodromal stage; (**e**) positive engagement in the acute stage; (**f**) negative engagement in the acute stage; (**g**) numbers of comments in the acute stage; (**h**) numbers of shares in the acute stage; (**i**) positive engagement in the chronic stage; (**j**) negative engagement in the chronic stage; (**k**) numbers of comments in the chronic stage; (**l**) numbers of shares in the chronic stage.

Figure 5 lists the average numbers of engagement obtained in different stages by each post category, ordered from the highest to the lowest for every type of social media engagement.

#### 5.5.1. Prodromal Stage

In the prodromal stage, there were only three post categories (i.e., Plans and Measures, Public Health Messages and Rumor Control) found in the data because the local outbreak had not occurred. All three categories could retrieve moderate levels of positive engagement (Figure 5a) and shares (Figure 5d) in this stage, and their negative emotions and comments were relatively low in volume (Figure 5b,c). Below are some examples of posts with high positive emotions. Since the authorities did not know much about the virus in the early stage, these were mainly preventative measures and generic messages of maintaining personal hygiene:

“In response to clusters of pneumonia cases of unknown cause in Wuhan, the Health Bureau started today to screen passengers on flights from Wuhan at the Macao Airport. No abnormalities were found for the time being… [Link]”

(Category: Plans and Measures; posted on 1 January 2020).

“To prevent the pneumonia of unknown cause in Wuhan, everyone should pay attention to personal hygiene. When coughing or sneezing, cover your mouth and nose with a tissue. If you don’t have a tissue, you can cover your mouth and nose with your elbow or upper sleeve… [Infographic]”

(Category: Public Health Messages; posted on 16 January 2020).

#### 5.5.2. Acute Stage

In the acute stage, the engagement of users focused on the areas of Appreciation, Community Resilience and information updates such as Press Conference Live and Rumor Control. Particularly, Appreciation recorded the largest volume of positive emotions and the number of shares (Figure 5e,h); whereas Community Resilience also gained a high level of positive emotions (Figure 5e) and notably zero negative emotions (Figure 5f). Rumor Control attracted the second-high number of shares (Figure 5h). Additionally, Press Conference Live and Appreciation topped the numbers of comments in Figure 5g, suggesting that substantial discussions occurred under these types of posts in this stage. Below list some samples of the content that people engaged with most, which are generally about how everyone plays their parts to control the outbreak.

“Thanks to our medical staff—In the difficult time of epidemic prevention and control, not only in hospitals, but also in health centers, blood donation centers and other locations, medical staff are doing their best to continue providing services to Macao residents. We would like to say thank you! … [Photos]”

(Category: Appreciation; posted on 31 January 2020).

“In the afternoon on 4 February, [an entrepreneur] donated 100,000 surgical masks to the Macao SAR government to help prevent and control the epidemic and reduce the risk of virus transmission. [Photos]”

(Category: Community Resilience; posted on 5 February 2020).

“There is a rumor that the Health Bureau will distribute face masks to Macao residents free of charge. The Coordination Center clarified again today that masks need to be purchased at their own expense… Meanwhile, we urge not to spread false information.”

(Category: Rumor Control; posted on 23 January 2020).

However, in the acute stage, we observe that categories Latest News and Rumor Control showed a higher level of negative emotions (Figure 5f). The content of these posts was mainly related to the reporting of new COVID-19 cases. Here, lists one example: “There are three new imported cases of new coronavirus infection in Australia, increasing to 5 cases in total. The third newly imported case of novel coronavirus infection is a female…” (Category: Latest News; posted on 26 January 2020).

#### 5.5.3. Chronic Stage

In the chronic stage, similar to the previous stage, Appreciation and Community Resilience remained the top two categories in terms of positive emotions (Figure 5i), and Press Conference Live and Appreciation posts were still commented by the most number of users (Figure 5k).

“The Public Security Police Force continues to assist customs clearance and help travelers to create their health QR codes at various ports... [Photos]”

(Category: Appreciation; posted on 11 July 2020).

“The Education and Youth Affairs Bureau collects epidemic prevention materials for schools donated by enterprises to schools — [a business] donated 10,000 masks, 54 disinfection supplies and 1200 chlorine disinfection tablets to Macao schools on August 10... [Photos]”

(Category: Community Resilience; posted on 13 August 2020).

Meanwhile, Public Health Messages obtained the highest number of shares (Figure 5l). As shown below, these posts regarded mental health support, information about getting back to the normal, and urging the public to remain vigilant for the virus.

“In response to the epidemic, the Department of Social Work of the Macao Polytechnic Institute used their professional strengths and launched the [course name] online lecture free of charge. They, hope to provide assistance to residents who need physical and mental support... [Link]”

(Category: Public Health Messages; posted on 4 May 2020).

“The Macao SAR Government appeals for washing hands, wearing masks, keep social distancing and not gathering... [Infographic]”

(Category: Public Health Messages; posted on 30 July 2020).

Finally, Plans and Measures and Press Conference Live attracted more negative emotions in the chronic stages (Figure 5j). These posts were mainly related to the violations of epidemic controlling laws and the minor issues in the government’s arrangements of COVID-19 measures.

“A person who was under quarantine in a designated hotel room left the room to another one on the same floor without authorization. The Macao Government Tourism Office has condemned the behavior and instructed hotels to strengthen the security. [Other measures…] Residents are reminded that they must abide by the relevant laws...”

(Category: Plans and Measures; posted on 29 July 2020).

“New Coronavirus Infection Response Coordination Center Press Conference (27/08) [about the arrangements of backing to school] [Video]”

(Category: Press Conference Live; posted on 27 August 2020).

### 5.6. Word Frequency Analysis of Comments

We applied a word frequency analysis in order to explore the popular concerns that emerged from the comments of government social media during COVID-19. Table 7 lists the most common 10 words that appeared in user comments of the posts in three stages and their numbers of occurrences. In the prodromal stage, most comments discussed the preventive measures enforced by the government and the public showed a positive attitude towards these measures. In the acute stage, three themes can be identified, including: (1) questions about the government’s operations and hoping the reporters at the pressers pursue answers; (2) cheering up others and appreciations to front-line workers; (3) border closure and quarantine. In the chronic stage, the topics of comments shifted to questions about daily lives, for example, education (denoted by words such as children and students), public transportation (e.g., the word bus) and face masks. In all stages, we observe that users mentioned other neighboring locations such as Hong Kong and Gongbei (which is a small region in Southern China adjacent to Macao) in many comments.

## 6. Discussion

This research empirically shows how the trend of engagement with government social media changes throughout pandemics, that is, it is lower in both the prodromal and chronic stages and higher in the acute stage. The public also demonstrates their interests in different topics in different stages, as well as their intentions for interacting with governments and voicing their concerns. This section elaborates on these aspects and discusses the potential applications of government social media in future outbreaks.

### 6.1. Principal Results

In response to RQ1, we identified that the engagement on the government social media was lower at the beginning, then peaked at the acute stage in which the disease was widely transmitted. This observation applies to most of the post categories, and can be explained with situational relevance, that is, people tend to obtain health information when they perceive strong relevance to the situation [64]. In the prodromal stage, the city had not struck by the virus which may cause the public to have a “wait and see” attitude, resulting in a drop in engagement on social media at the earlier time. After the acute stage, as the pandemic started to ease, we could see the trend of engagement decreased over time. This may reflect that the public is less sensitive to the regular updates, and they tend to focus on other areas as their lives get back to normal.

Answering RQ2, the shift of focuses of the engagement is evident from our context analysis. In the prodromal stage, the engagement largely concentrates on the government’s measures and the prevention of the disease, which is in line with other research, suggesting the seeming importance of preventive measures [65,66,67]. In addition, reflected in the correlations, positively perceived messages are likely to be shared by users in this stage, implying that the public is willing to further disseminate information provided that it is positive. With hindsight, the measures for fighting pandemics require everybody to be involved, therefore, the use of government social media needs to retain a positive tone, meanwhile it can be strengthened to increase the vigilance of the public and the awareness of preventive measures, such as hand washing, maintaining personal hygiene, social distancing, in the early phase of future outbreaks.

Then, in the acute stage, the public overwhelmingly reacts to posts about rumor control, community resilience and appreciation. As such, governments are recommended to use more such messages to bring communities together to combat diseases. On the other hand, while the posts providing news and updates (e.g., case reports) are often negatively engaged, their engagement remains at a high level. In addition, the word frequency analysis of comments shows that both prevention and controlling measures are the main concerns in this stage. Therefore, in line with the literature in crisis management [20,68], the use of government social media in the acute stage should focus on providing up-to-date information and increasing transparency, regardless of the positive or negative nature of the content. The higher degree of sharing in the acute stage also indicates the potential of social media for disseminating information in a large scale, making it an effective tool for governments and authorities to broadcast updates during public health crises.

When the pandemic enters the chronic stage, the focus moves towards mental health support and the issues about daily activities. As a micro-economy, Macao has suffered a strong economic impact in its gaming [23,69] and hospitality [29,70] industries, while the city has enforced stringent measures in the areas which are comprised of its main income, for instance, closing down casinos, the mandatory quarantine of travelers and the ban of tourists. Many places in the world actually face the same problem. Unlike the previous outbreak of SARS, this pandemic has lasted a much longer duration, therefore people have gradually become anxious under this situation. As the COVID-19 crisis eases, it is understandable that the public turns their focuses to the return of the normal. As a practical implication, it is useful for governments to adopt social media to provide mental health support and information about the recovery of business and economy, in order to reduce the uncertainly of the public and to plan out the directions for the policies after the chronic stage.

By analyzing the comments, we argue that users demonstrate a desire to interact with governments and provide feedback using the commenting feature of social media. For example, as shown in the results, the public considered some preventive measures good in the prodromal stage, and viewers asked questions during online press conferences in other stages. Comments can be seen as a type of engagement with a higher cognitive load on government social media [9]. In this case, as the public is still keen to make comments despite the cognitive load, it may represent their demand of greater participation in policymaking and policy execution. As another implication, government social media can act as a probe for governments to receive feedback and adjust their measures in the course of pandemics.

Although digital media including social media is ubiquitous in the pandemic, there is a lack of formalized guidance for their use. To address this gap and answer RQ3, we propose practical implications for using government social media in three specific areas: rumor control, regular updates and community cohesion.

### 6.2. Rumor Control

Highlighted by many scholars, the infodemic is a part of this COVID-19 pandemic as misinformation is wildly spread on the internet worldwide [2,3,71]. Being a part of this global phenomenon, Macao is not immune to the infodemic and therefore the government posted a wide range of posts in order to control rumors. Fortunately, these rumor controlling posts could keep the engagement with the public with a moderate level of sharing in all crisis stages, which further reflects the need for trustworthy updates. Supported by necessary legislation (e.g., Article 26 in the Macao Civil Protection Law), government social media can help the authorities to detect anomalies such as rumors and misinformation. After rumor detection, in order to clarify and correct those rumors, government social media will be one of the channels to amplify the correct messages among the public. Our findings reinforce the potential of using government social media to counteract falsehood information.

### 6.3. Regular Updates

Starting on the day of the first confirmed case, the Macao SAR government had been organizing daily press conferences and these were aired live on social media simultaneously every day. This is a necessary step for improving the transparency of governments and reducing the uncertainty of the public during this COVID-19 crisis [4]. Not many places in the world organized official daily press conferences in the pandemic. However, in some locations with a success for controlling the virus (for example, Taiwan and Melbourne), daily and persistent press conferences were considered as a welcomed measure [72,73]. In the case of Macao, it can be seen that these press conference live posts can maintain a constant level of engagement in the acute stage, which shows the usefulness of government social media in providing regular updates. In addition, as shown in our examples of posts, most of the regular updates authored by the Macao government include pictures, infographics or videos. These images and videos increase the interactivity of posts, which can help the audience to easier navigate and understand health information [74], and may attract more users to share them on social media platforms [47]. As such, authorities should also focus on producing and using appropriate multimedia along with text-based content.

### 6.4. Community Cohesion

Information about community resilience and appreciation can attract steady and overwhelming positive engagement with minimal negative responses. Research suggests that the quick involvement of the community can help with the control of COVID-19 and protect the interests of the public [75]. In fact, community organizations often work with the Macao SAR government in different areas, including the recovery of natural disasters and this pandemic [25,76]. Social media posts allow the public to understand the community efforts spent in the outbreak. Echoing the call from Yang and Su [77], we argue that government social media can be used for creating a positive sentiment against the pandemic and bringing communities together.

In addition, other research highlights that government social media often carries only one-way communication in disease outbreaks [17,20], that is, messages are conveyed from governments to the public. Our data show that the public responded by leaving comments, which can be used as indicators for measuring the acceptance of certain policies in the pandemic and for fine-tuning them to meet the needs of people. However, such citizen–government interactions still remain a one-way communication, and there is a potential to turn this into a bidirectional interaction for maximizing its impact. By responding to individual comments, governments can build an image of openness and take this opportunity to explain and clarify certain policies and measures.

### 6.5. Limitations

There are cultural and political differences in the use of social media. Users may not be aware of the emotions other than “likes” and may not have the same understanding of different emotions provided by Facebook, which may introduce discrepancies when interpreting users’ reactions. Our content and word frequency analysis provided multiple perspectives of the data which helps to alleviate these issues. Additionally, different governments adopt different approaches to control COVID-19. As such, other researchers from other places may have different observations about government social media, and we suggest conducting localized research and generating collective knowledge in this discipline. Besides, classification errors might happen in the content analysis process. However, we used inter-coder reliability checks to ensure the error rate was acceptable for academic research. Our future work includes a deeper examination of Facebook data using computational analytics to address some of these issues.

## 7. Conclusions

In this paper, we studied how government social media could engage with the public using the lens of the staged crisis management model and examined how government social media was used in the COVID-19 pandemic. Our work has found that the engagement of government social media is associated with crisis stages: it is lower at the prodromal stage, then surges in the acute stage, and gradually decreases in the chronic stage. In terms of the content, the public changes their focuses from the pandemic to the recovery (such as policies and support) after the acute stage. The engagement of other categories of content declines over time. In different stages, government social media should be aligned with the specific content of those stages. For the use of government social media, we propose that it is a useful tool for informing the public and interacting with communities. In addition, it can play a role in rumor control and fostering community cohesion. We hope that our findings can prepare authorities for using social media in fighting future outbreaks of infectious diseases.

## Figures and Tables

**Table 1:** Main measures and actions taken by the Macao SAR government.

Policy Category	Measures and Actions
Disposition of government	Established the COVID-19 Coordination Center Increased the alert level of public emergencies Improved public education about the virus Global procurement of face masks
Prevention and control	Strengthened health screening and quarantine Reduced social gatherings Shut down casinos, businesses and entertainment premises Suspended classes in schools and universities Mandated the use of face masks on public transport Set up an online personal health declaration system (Health QR Code)
Medical support	Suspended non-critical medical services Provided support to front-line medical staff Provided mental health assistance
Border control and immigration	Limited travelers’ entry to Macao Suspended self-service customs clearance Shortened customs clearance time to avoid queues and allow social distancing
Business support	Established a special anti-epidemic assistance fund Launched economic assistance measures Tax reduction and exemption Provided subsidized employee training
Livelihood support	Issued electronic shopping vouchers Subsidized water and electricity bills Monitored the prices of food and other daily necessities

**Table 2:** Crisis stages of the COVID-19 outbreak.

Crisis Stage	Definition	Start of Stage	End of Stage
Prodromal	This comes before the actual crisis, and its main focus is to prevent or delay the crisis from happening.	The start of the current analysis	The first confirmed COVID-19 case
Acute	This stage follows the prodromal stage. It is signaled by the sudden onset of the event, and the event often develops rapidly. The main goals lie in controlling the crisis and avoiding its deterioration.	The first confirmed case	28 days after the last case of local transmissions ^a^
Chronic	The crisis situation begins to ease in this stage and its appearance is less dramatic in appearance. As such, the focuses should be on relieving controlling measures, reducing damage and initiating the steps towards recovery.	The first day after the acute period	The end of the current analysis
Crisis Resolution	The crisis is over in this stage. Learnings should be synthesized for preparing the responses to future crises and the society/organization is returning to normal.	Not applicable (since the world is still in the middle of the pandemic at the time of writing, this stage is not applicable).	

^a^ The end of an outbreak can be declared when there are no infections after two incubation periods (28 days for SARS-CoV-2) as commonly used in other research [35,36].

**Table 3:** Categories of COVID-19 social media content in the existing literature.

Category	Concepts and Related Literature
Plans and Measures	Disposition of government [48] Government’s handling of crisis [9] Preventive measures [8] Government response [12,49] Policies, guidelines and official actions [17] Notifications and measures been taken [50] Control measures [51]
Public Health Messages	Civic skills [41] Caring of self-interest [48] Education [18] Disease prevention [16] Popularization of prevention and treatment [49]
Rumor Control	Rumor Control [41] Falsehood correction [8] Counter-rumor [50]
Latest News	Latest news [9] New cases of COVID-19 [15]
Appreciation	Appreciation of front-line workers [9] Appreciation [8]
Community Resilience	Donations of money, goods or services [50] Making donations [49]

**Table 4:** Date and week ranges of the three crisis stages in Macao.

Crisis Stage	Date Range	Week Range (of Calendar Year 2020)
Prodromal	1 January 2020–21 January 2020	Week 1–4
Acute	22 January 2020–25 April 2020	Week 4–17
Chronic	26 April 2020–31 October 2020	Week 18–44

**Table 5:** Descriptive statistics of the engagement in three stages.

	Prodromal (N = 18)				Acute (N = 481)				Chronic (N = 1165)			
Metrics	Mean	SD	Min	Max	Mean	SD	Min	Max	Mean	SD	Min	Max
Positive Emotion	35.39	60.56	5	262	99.59	372.89	0	4504	20.29	42.48	0	471
Negative Emotion	1.72	4.40	0	16	9.64	66.54	0	1267	2.24	23.01	0	667
Comments	2.72	9.36	0	40	34.83	124.30	0	2000	3.80	20.18	0	459
Shares	22.17	50.21	0	208	15.91	47.01	0	577	3.82	14.28	0	246

**Table 6:** Categories of posts derived from the Macao SAR Government Facebook data.

Name	Definition	Count	Example
Plans and Measures	Government’s plans and measures to combat the pandemic	542	“Starting from 0:00 h on 19 March all foreign worker ID card holders will be prohibited from entering Macau, except for those with a resident status of Mainland China, Hong Kong and Taiwan… [Infographic]”
Public Health Messages	Messages for educating the public, persuading them to change behavior and preventing COVID-19 infection	99	“The representative of the Health Bureau explained that aerosol transmission is not equivalent to air transmission; aerosols are small droplets formed when patients cough, sneeze or talk to people…”
Rumor Control	Posts for rumor control or clarifying untrue information	22	“Regarding rumors on the internet that influenza vaccination can prevent COVID-19, the Health Bureau emphasized that the rumors are not true and appealed the public not to believe or spread false information…”
Latest News	Latest update about the pandemic, including regular updates of numbers	709	“In the past 24 h, there have been no new confirmed cases of COVID-19 infection in Macao, and a total of 10 confirmed cases have been maintained. All of which are in a mild condition. As of 3 pm, there are a total of 316 suspected cases in Macao...”
Appreciation	Appreciation message to front-line workers and public staff for controlling the pandemic	44	“In response to the anticipated increase in tourists, all relevant departments in Macao have made specific arrangements, strengthened epidemic preventive work and passenger management. Let’s have a look at what they do and thank them for their efforts.”
Community Resilience	Actions (e.g., donations of resources) taken by communities (e.g., non-government organizations and individuals) to combat the pandemic	17	“ [The name of an enterprise] donated 4000 L of disinfectant alcohol to the SAR government…”
Press Conference Live	Live video of the government’s press conferences about the pandemic and its latest development	231	“Press conference on the latest outbreak of COVID-19 and announcements of various prevention and control measures in Macao [Date] [Video]”

**Table 7:** The most used 10 words in user comments (word frequencies shown in brackets).

Rank	Prodromal Stage	Acute Stage	Chronic Stage
1	achieve (3)	question (379)	resident (109)
2	good (2)	Hong Kong (340)	Hong Kong (88)
3	prevent (2)	border closure (338)	children (79)
4	measure (2)	Macanese (295)	student (63)
5	border entry (2)	border entry (279)	quarantine (59)
6	well (2)	quarantine (276)	why (59)
7	quarantine (2)	cheer up (265)	question (55)
8	Gongbei (2)	appreciate (257)	teacher (45)
9	check (2)	Portuguese reporter (252)	face mask (43)
10	not bad (2)	reporter (249)	bus (40)

## Data Availability

The data presented in this study are available on request from the corresponding author. The data are not publicly available due to the restrictions of the social media platform.
